# Drug-induced heart failure: a real-world pharmacovigilance study using the FDA adverse event reporting system database

**DOI:** 10.3389/fphar.2024.1523136

**Published:** 2025-01-15

**Authors:** Youqi Huang, Xiaowen Chen, Mingyu Chen, Yuze Lin, Bingqi Chen, Hongjin Gao, Min Chen

**Affiliations:** ^1^ Shengli Clinical College of Fujian Medical University, Department of Pharmacy, Fujian Provincial Hospital, Fuzhou University Affiliated Provincial Hospital, Fuzhou, China; ^2^ College of Pharmacy, Fujian Medical University, Fuzhou, China; ^3^ Department of pharmacy, Xiamen Medical College, Xiamen, China

**Keywords:** heart failure, adverse events, pharmacovigilance, FAERS, data mining

## Abstract

**Objective:**

Although there are certain drug categories associated with heart failure (HF), most of the associated risks are unclear. We investigated the top drugs associated with HF and acute HF (AHF) reported in the FDA Adverse Event Reporting System (FAERS).

**Methods:**

We reviewed publicly available FAERS databases from 2004 to 2023. Using the search terms “cardiac failure” or “cardiac failure acute” and classifying cases by drug name, we processed and analyzed drug reports related to HF or AHF.

**Results:**

From 2004 to 2023, 17,379,609 adverse drug events were reported by FAERS, of which 240,050 (1.38%) were reported as HF. Among those with HF, the male-to-female ratio was 0.94% and 52.37% were >65 years old; 46.2% were from the United States. There were 5,971 patients with AHF. We identified 38 drugs and 13 drug classes with a potential high risk of causing HF, and 41 drugs and 19 drug classes were associated with AHF. The median onset times of HF and AHF were 83 days (IQR: 11–416) and 49 days (IQR: 8–259), respectively. The Weibull shape parameter (WSP) test showed early failure-type profile characteristics.

**Conclusion:**

This study highlights key drugs associated with drug-induced HF and AHF, emphasizing the importance of early risk assessment and close monitoring, particularly during the initial stages of treatment. These findings contribute to a better understanding of drug-induced HF and provide a basis for future research on its underlying mechanisms.

## 1 Introduction

Heart failure (HF) is a complex syndrome caused by a variety of factors, including abnormal changes in cardiac structure or function and ventricular systolic or diastolic dysfunction (Packer, 2018; Bozkurt et al., 2021). Its potential causes include ischemic heart disease, coronary heart disease, diabetes, obesity, tachycardia, and the negative effects of certain drugs, such as drug toxicity and resistance (Packer, 2018; Bozkurt et al., 2021; Heidenreich and Sandhu, 2024). Its prevalence is rapidly increasing worldwide and it is strongly associated with high morbidity and mortality (Ziaeian and Fonarow, 2016; Borlaug, 2020; Greene et al., 2023). HF is a leading cause of hospitalization and death, particularly in the elderly population (Cho et al., 2023). In the United States, the national health and nutrition examination survey estimates that more than 5.1 million (≥20 years old) or more suffer from HF (Go et al., 2014). Predicts 2030, the number will increase 46%, reaching more than 8 million people (Heidenreich et al., 2013). As the global population ages, the health and economic burdens of HF on the elderly is increasing. Despite continuous advancements in medical diagnoses and treatment strategies, the mortality and rehospitalization rates of patients with HF remain high, and their quality of life is low (Qin et al., 2018).

Despite preclinical safeguards against HF induced by drug administration, postmarketing surveillance remains crucial because certain cardiotoxic agents may evade initial review. Historical data indicate that drug-induced cardiac toxicity, particularly in HF, tends to be underestimated, with incidence rates ranging from 11.0% to 20.0% (Destere et al., 2024). Increasingly, it is recognized that drug-induced HF is a significant but often overlooked cause of heart problems. Diagnosing drug-induced HF remains challenging due to the lack of recognized clinical, biochemical, and radiological markers that can clearly distinguish it from other causes of HF, necessitating the exclusion of other common causes. While it is well known that many drugs, including anthracyclines, traditional chemotherapy agents (such as cyclophosphamide, cisplatin, and paclitaxel), and antiarrhythmic drugs, can induce or worsen HF, identifying new drugs associated with HF still requires diligent postmarketing surveillance (Maxwell and Jenkins, 2011; Diaz et al., 2024).

The FDA Adverse Event Reporting System (FAERS) reports drugs associated with adverse drug reactions. However, determining the exact causal relationship between individual drugs and adverse events (AEs) can be challenging. Consequently, FAERS is limited in drawing firm conclusions about the prevalence, incidence, and causality of adverse drug reactions (ADRs), and there can be significant reporting bias based on national concerns or regional awareness. Despite these limitations, FAERS contains a vast number of drug-related AEs. We conducted a comprehensive analysis of the FAERS database to identify drugs associated with HF. In this study, we focused on the top 50 drugs most frequently reported in association with HF, which were subsequently classified into pharmacological or therapeutic subgroups.

In addition, HF can be categorized into chronic HF (CHF) and acute HF (AHF) based on the onset and progression of symptoms. CHF is a long-term condition, often caused by underlying diseases such as coronary artery disease, hypertension, or diabetes, and is characterized by progressive symptoms and ventricular remodeling. In contrast, AHF represents a sudden onset or worsening of symptoms, often triggered by acute events such as myocardial infarction, arrhythmias, or severe infections, and is associated with higher rates of hospitalization and mortality (Chioncel et al., 2019; Arrigo et al., 2020; McDonagh et al., 2021). Thus, we also performed subgroup analyses of AHF and assessed how trends have changed over time.

## 2 Methods

### 2.1 Data source

The data for this study were from the FAERS database, the FDA’s post-market safety-monitoring program for all marketed drugs and therapeutic biologics, which contains AE reports submitted by healthcare professionals, consumers, and manufacturers. We conducted a pharmacovigilance study of post-marketing drug-induced HF using these data from Q1 2004 to Q4 2023. We reviewed and analyzed all HF reports recorded.

### 2.2 Identification of target AEs

ADR information was coded into standardized medical terms called preferred terms (PTs) using the Medical Dictionary for Regulatory Activities (MedDRA). Standardized MedDRA Queries (SMQs), a built-in tool in MedDRA consisting of a series of PTs indicating similar medical conditions, are designed to aid in retrieving cases of interest from the MedDRA coded database and to optimize ADR signal detection and evaluation. There are two types of searches to identify target cases, namely, broad-scope and narrow-scope searches. The former includes all conditions or PTs in the field, while the latter focuses only on conditions or PTs closely related to specific interests to ensure the specificity of AE recognition (Li et al., 2023). Reference MedDRA 26.1, this research only select “cardiac failure (SMQ)” PTs to identify targets in the narrow-scope search AE report (Supplementary Table S1).

### 2.3 ADR signal detection method

Disproportionality analysis is a data mining method commonly used for the detection of ADR signals. The idea is to compare the frequency of the target drug event with the background frequency. In a database containing all drug event reports, when the target drug event combination (DEC) occurs significantly more frequently than the background frequency of other drugs in the entire database and reaches a set threshold, a signal is considered to have been generated. In this study, the reporting odds ratio (ROR) method was used for data analysis within the disproportionality analysis framework. The four-grid table and algorithm used in calculations are shown in Supplementary Table S2. When the lower limit of the 95% CI of the ROR value is > 1 and at least three target AEs are reported, the target drug is considered potentially high risk for leading to the target AE, generating a positive ADR signal.

### 2.4 Cumulative incidence of HF and time-to-onset

Time to onset was defined as the period from the initiation of medical therapy to the diagnosis of HF. Therefore, only reports with data on time of onset were analyzed. Onset time data were analyzed based on median, quartile, and the Weibull shape parameter (WSP) test. The rate of AEs after the initiation of treatment depends on the mechanism of action of the drug and generally varies over time. By contrast, AEs not related to drug therapy tend to occur at a constant rate. The WSP test can determine the rate of change in the incidence of AEs (Kinoshita et al., 2020). The scale parameter α of the Weibull distribution determines the scale of the distribution function; a larger value stretches the distribution whereas a smaller one compresses it. The shape parameter β determines the shape of the distribution function, where a larger value gives a left-skewed curve and a smaller one produces a right-skewed one. The β was used to represent the hazard function without a reference population as follows: at β < 1 and a 95% CI < 1, the hazard was considered to have decreased over time (early failure profile); when it was equal to or nearly 1 and its 95% CI included 1, it was estimated to occur constantly over time (random failure profile); and when it was >1 and its 95% CI excluded 1, it was considered to increase over time (wear-out failure profile).

### 2.5 Data acquisition, processing, analysis, and summary

**FIGURE 1:**
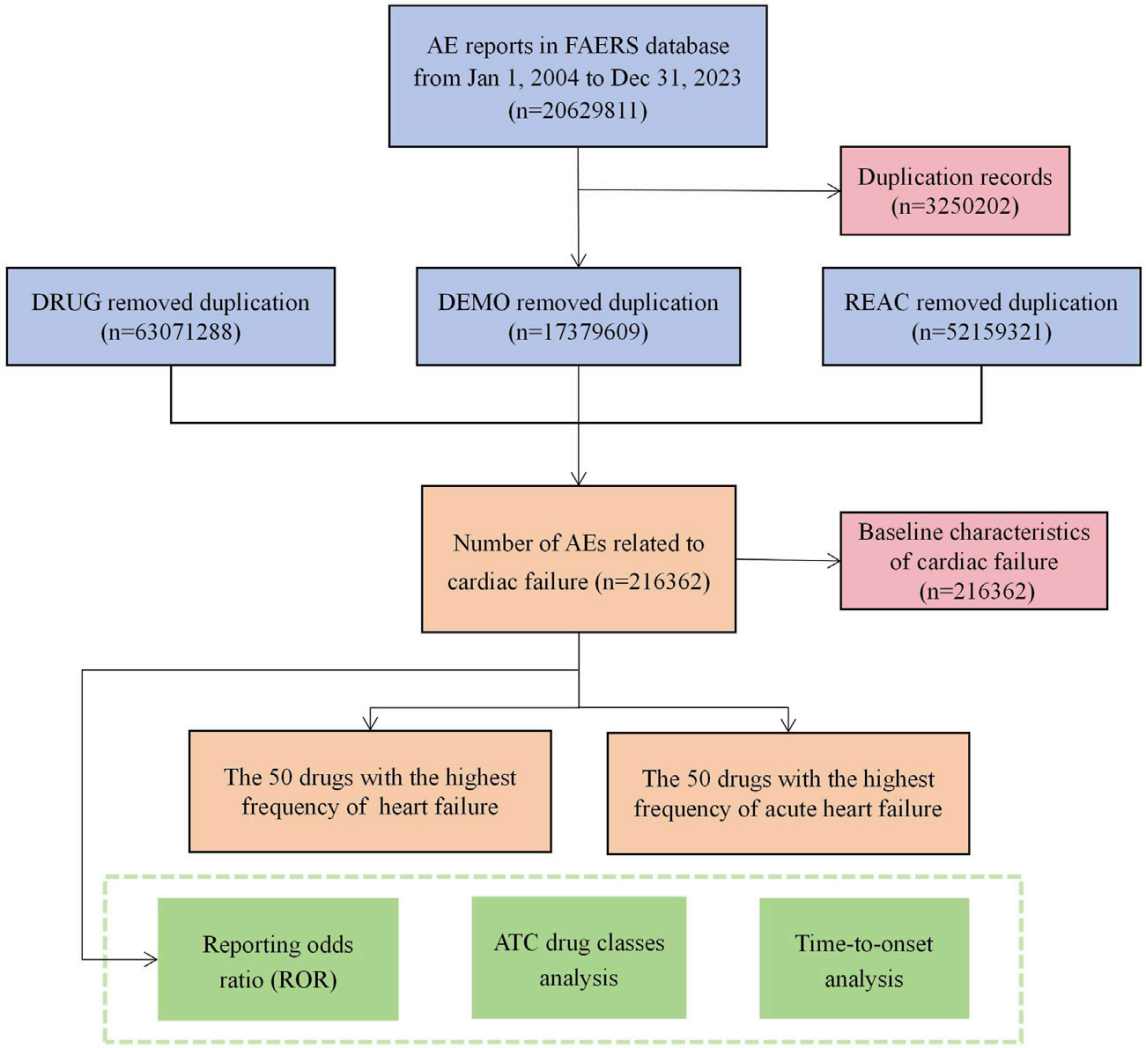
Flowchart of potential culprit-drug identification. (DEMO demographic and administrative information, DRUG drug information, REAC preferred terminology for adverse event, PS primary suspect drug).

In all, 20,629,811 reports were obtained from the FAERS database during the study period. First, because the database is updated regularly, the data had to be analyzed to remove any redundant instances that were previously publicly reported. To this end, a data deduplication procedure was performed according to the guidelines provided by the FDA prior to statistical analysis. To accomplish this, the most recent FDA_DT was chosen when the CASEID values were identical, and higher PRIMARYID values were selected when CASEID and FDA_DT matched (Shu et al., 2022). When duplicate, incomplete, and incorrect reports were excluded, the total number of reports was reduced to 17,379,609. The specific flow chart is shown in Figure 1.

Second, AE reports were retrieved using the PTs in Supplementary Table S1 to identify all reports related to HF received by FAERS from 1 January 2004 to 31 December 2023. The basic information of patients and reports was collected and summarized, including reporting year, reporting country, author, patient age, patient sex, patient outcome, and more.

Third, from the collection of major suspected PS drugs, we excluded ambiguous drug names and integrated drugs with the same composition. Then the top 50 drugs associated with HF were identified. At the same time, the anatomical therapeutic chemical (ATC) classification system was used to code the preliminary drug list to obtain ATC drug classes (second ATC level) containing the top 50 drugs.

Fourth, according to the final drug list, the ADR signals of each drug and ATC drug classes were detected. Then, according to the ADR signal detection results, the distribution characteristics of the drug ADR signals were summarized.

Finally, we analyzed time of onset and identified AHF associated wtih PTs (acute left ventricular failure, MedDRA code: 10063081; acute right ventricular failure, MedDRA code: 10063082; cardiac failure acute, MedDRA code: 10007556). R version 4.3.2 was used for data acquisition, processing, and analysis.

## 3 Results

### 3.1 Baseline characteristics of patients with HF

The baseline characteristics of patients with drug-related HF are shown in Table 1 and Supplementary Figure S1. From the first quarter of 2004 through the fourth quarter of 2023, data on a total of 216,362 HF patients were reported. Of this sample, 100,658 (46.5%) were women, 94,264 (43.6%) were men, and 21,440 (9.9%) did not indicate their sex. In terms of age composition, most patients were in the 65–85 years age group, accounting for 32.5% of the sample. The highest number of AEs was reported in the United States (46.2%), followed by Japan (6.9%), France (5.1%), Canada (3.9%), and Germany (3.8%). The annual numbers of AEs reported are shown in Supplementary Figure S1, in which 2015 is the year with the largest number of reports. With regard to reporting sources, physicians were the main submitters, responsible for 33.8% (Supplementary Figure S1) of reports. Death or life-threatening events were reported in 63,442 (29.3%) and 14,422 (6.7%) cases, respectively. Hospitalization (40.7%) was the most frequently reported serious medical event (Supplementary Figure S1).

**TABLE 1:** Clinical characteristics of reports from the FAERS database (2004–2023).

Characteristics	Drug-related heart failure (N = 216,362)
Number	216,362
Sex	
Male	94,264 (43.6%)
Female	100,658 (46.5%)
Unknown	21,440 (9.9%)
Age	
<18	4,376 (2.0%)
18 ≥ and <65	66,722 (30.8%)
65 ≥ and <85	68,625 (31.7%)
≥85	9,535 (4.4%)
Unknown	67,104 (31.0%)
Weight	
<50	7,004 (3.2%)
50 ≥ and <100	44,470 (20.6%)
>100	11,190 (5.2%)
Unknown	153,698 (71.0%)
Occupation of the reporter^a^	
Healthcare professional	134,873 (62.3%)
Non-healthcare professional	70,687 (32.7%)
Unknown	10,802 (5.0%)
Country of the reporter (Top six)	
United States	99,942 (46.2%)
Japan	14,919 (6.9%)
France	11,016 (5.1%)
Canada	8,457 (3.9%)
Germany	8,137 (3.8%)
United Kingdom	6,939 (3.2%)
Others	66,952 (30.9%)
Outcomeb	
Hospitalization	88,164 (40.7%)
Death	63,442 (29.3%)
Other Serious Outcome	42,172 (19.5%)
Life-threatening	14,422 (6.7%)
Disability	926 (0.4%)
Unknown	7,236 (3.4%)

^a^ Healthcare professionals including reporters such as physicians and pharmacists; non-healthcare professionals including reporters such as consumer and lawyer.

^b^ Because a case may experience different clinical outcomes during drug therapy, it is reasonable to expect that the sum percentage of the outcome under this item may exceed 100%.

### 3.2 Top 50 drugs associated with HF

**FIGURE 2:**
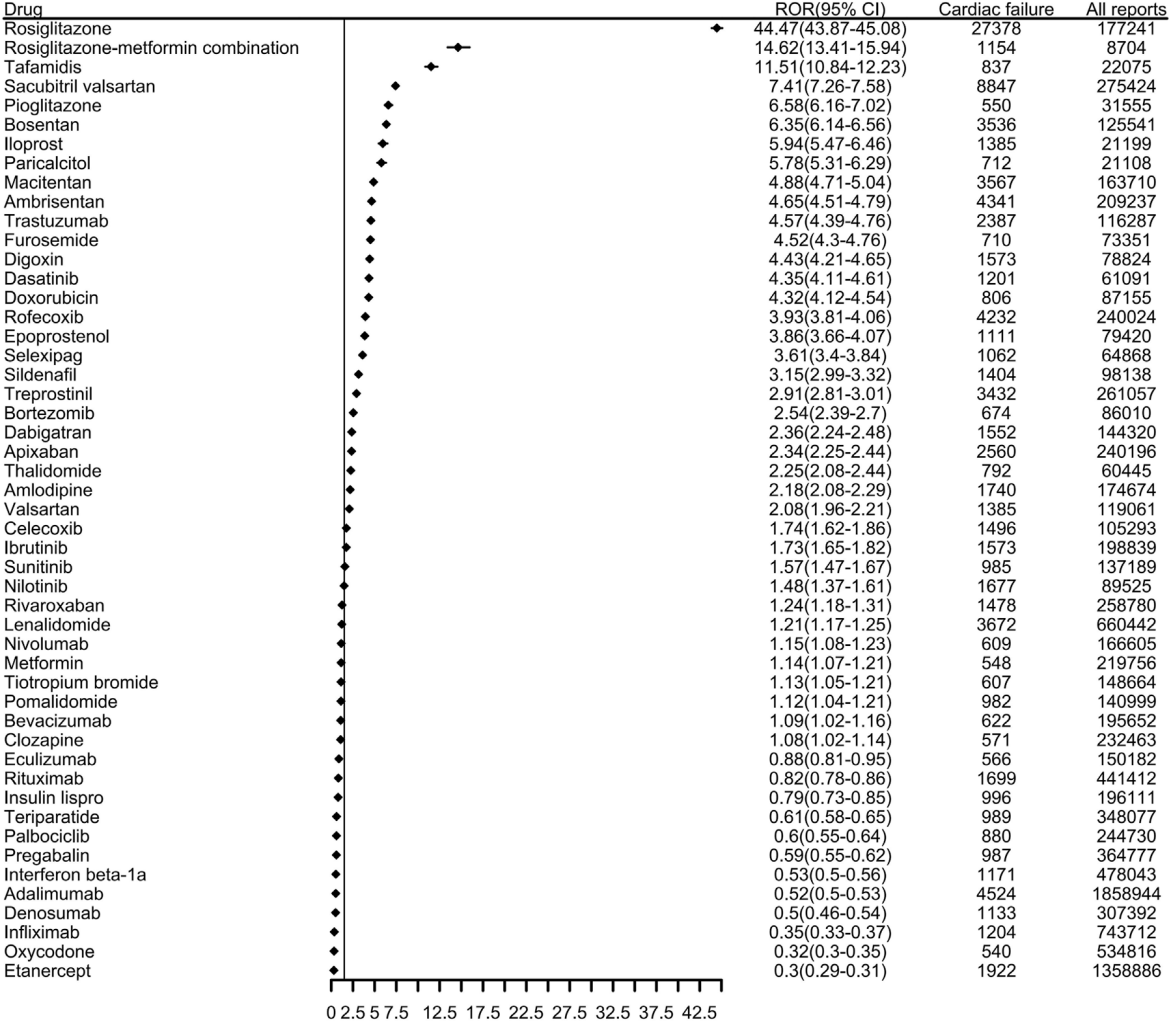
The top 50 drugs associated with reports of heart failure to the FDA from 2004 to 2023. (ROR, reporting odds ratio. CI, confidence interval).

The top 50 drugs related to HF reported to the FDA are shown in Figure 2 and Supplementary Table S3. These accounted for 110,359 HF-related events (46.0%) during the study period. Among them, the ROR signal test identified 38 as potentially high risk for causing HF; the number of AE reports and ATC codes are shown in Supplementary Table S3. More than 4000 HF-related events were associated with each of rosiglitazone, sacubitril valsartan, adalimumab, rofecoxib, and ambrisentan. Rosiglitazone in particular had many more reports than another other drug, at 27,378 HF-related adverse reactions; the ROR value was as high as 44.47.

### 3.3 Top 50 drug classes associated with HF

**FIGURE 3:**
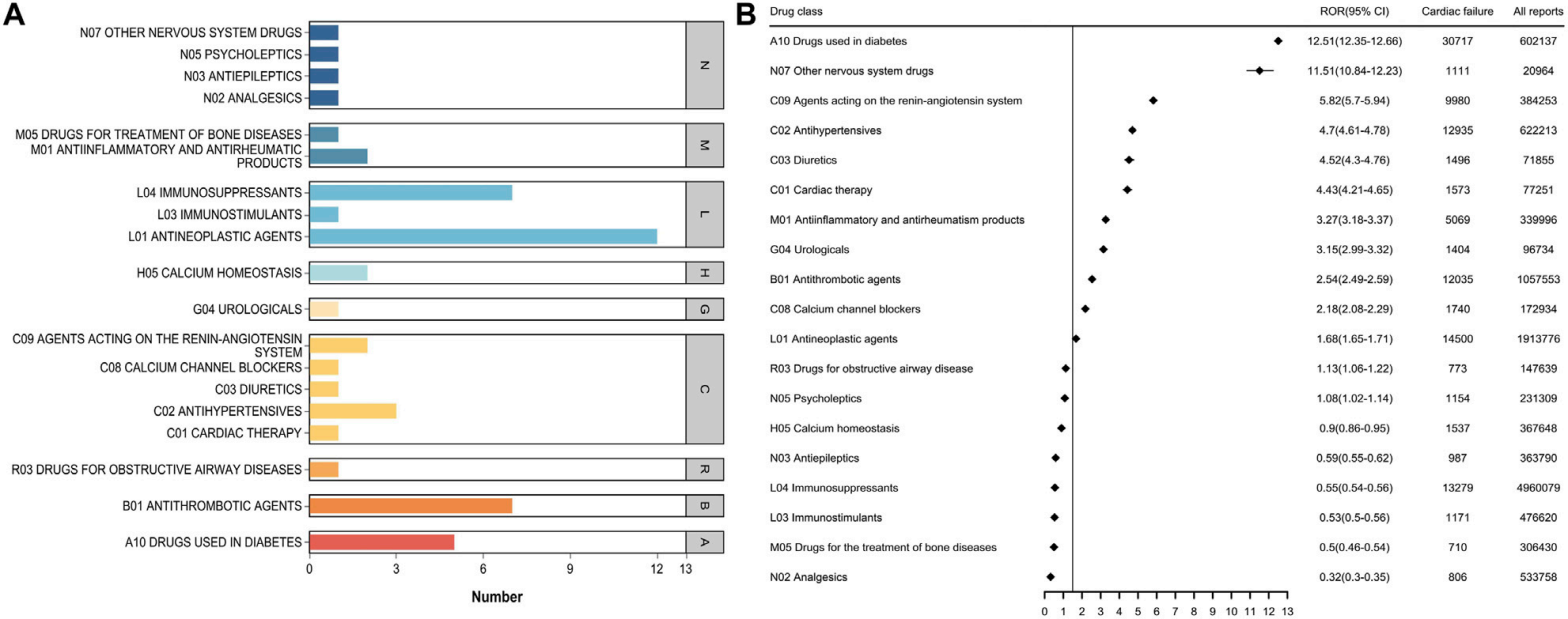
**(A)** Number of ATC categories for the top 50 drugs associated with heart failure report **(B)** ATC class signal values for the top 50 drugs associated with heart failure report.

When the top 50 drugs were grouped by ATC drug class (Figure 3A), the top five classes related to HF were antineoplastic agents, immunosuppressants, antithrombotic agents, drugs used in diabetes, and antihypertensives. Disproportionality analysis indicated that 13 drug classes including all of the aforementioned ones, plus agents acting on the renin-angiotensin system as well as antiinflammatory and antirheumatic drugs were associated with HF events (Figure 3B; Supplementary Table S4). Of these, drugs used in diabetes had the strongest association with HF, accounting for 12.80% of all HF-related events during the study period; they also had the highest positive signal value (ROR 12.51).

### 3.4 Top 50 drugs associated with AHF

**FIGURE 4:**
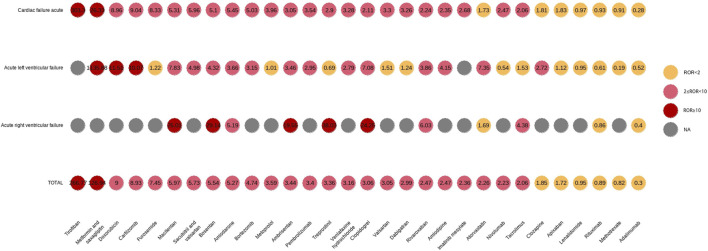
First 30 PT signal differences drugs in acute heart failure.

During the study period, the FDA received 6,072 reports of AHF, and the baseline characteristics of the patients are shown in Supplementary Table S5. Overall, AHF was rare, accounting for less than 0.04% of all AEs reported. We combined PTs associated with AHF and performed signal detection for the top 50 reported drugs (Supplementary Table S6). Including acute left ventricular failure (MedDRA code: 10063081), acute right ventricular failure (MedDRA code: 10063082), and cardiac failure acute (MedDRA code: 10007556). In total, 41 drugs were associated with AHF events, including tirofiban (ROR 266.77), metformin and saxagliptin (ROR 126.94), saxagliptin (ROR 43.91), doxorubicin (ROR 9.00), and carfilzomib (ROR 8.93), all of which had high positive signal values. In addition, subgroup analysis of these three PTs showed that the number of patients with AHF was higher, while the number of patients with acute right ventricular failure was lower. The signal differences among the top 30 drugs associated with the three PTs are shown in Figure 4.

### 3.5 Top 50 drug classes associated with AHF

**FIGURE 5:**
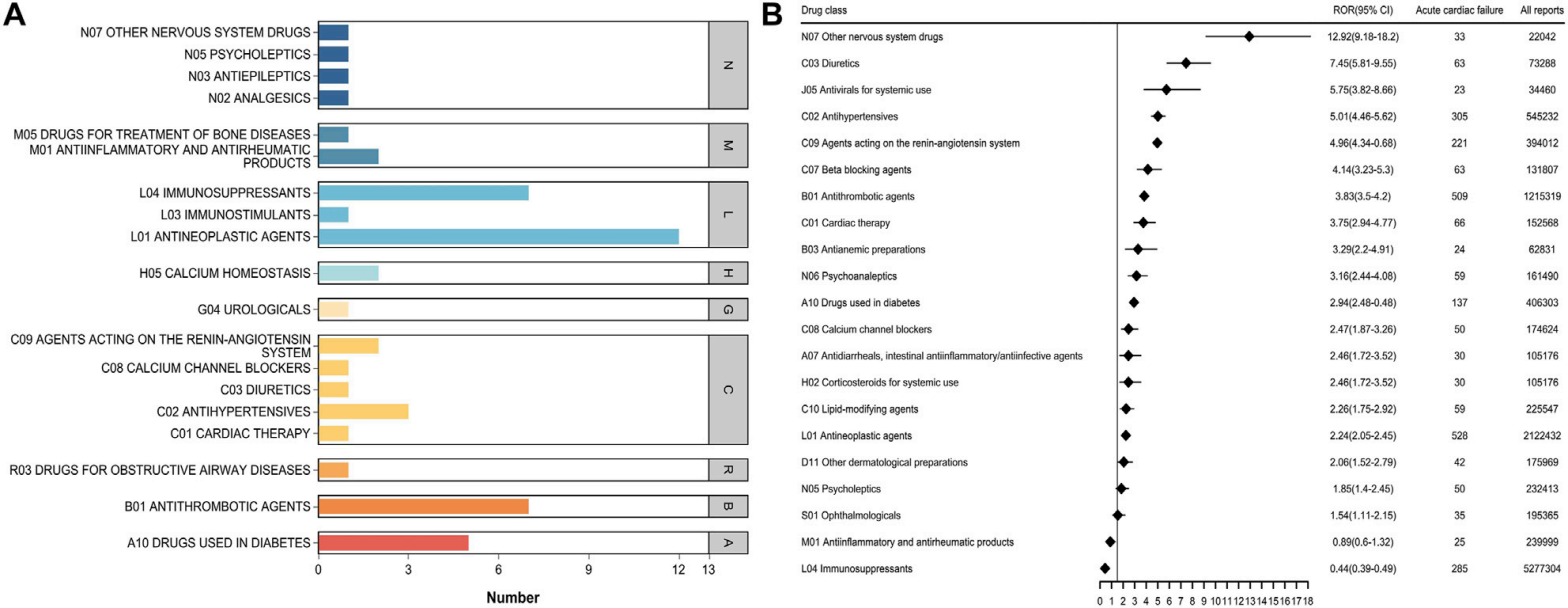
**(A)** Number of ATC categories for the top 50 drugs associated with acute heart failure report **(B)** ATC class signal values for the top 50 drugs associated with acute heart failure report.

The top 50 drugs associated with AHF were classified by ATC (Figure 5; Supplementary Table S7). There were 21 second-ATC levels, of which 19 were associated with AHF events. Other nervous system drugs (ROR 12.92) had the highest signal intensity, followed by Diuretics (ROR 7.45), Antivirals for systemic use (ROR 5.75), and Antihypertensives (ROR 5.01). Immunosuppressants were associated with a high number of AHF-related events but had a lower signal value. It is worth mentioning that the Other nervous system drugs category not only had a strong signal for HF-related AEs but also had a high positive signal value for the main drug classes in AHF.

### 3.6 Cumulative incidence of HF and time-to-onset

Of 216,362 and 5,971 reports of drug-related HF and AHF, 62,186 and 2,277, respectively, included data on time to onset, Supplementary Figure S2 shows the AEs onset time. The median time to onset was 83 days (IQR: 11–416) for HF and 49 days (IQR: 8–259) for AHF. More than 50% of reported cases occurred within the first 90 days after drug initiation. In WSP tests, the upper limit of the 95% CI for the shape parameter was <1 for both types of failure, indicating the presence of an early-failure profile, Table 2 summarizes the HF and AHF in WSP test results.

**TABLE 2:** Weibull shape parameter test for heart failure and acute heart failure.

Database	Case reports	Median (d) (25%–75%)	Scale parameter: α (95% CI)	Shape parameter: β (95% CI)	Type
Heart failure	62,186	83 (11–416)	191.02 (187.76–194.30)	0.49 (0.48–0.49)	Early failure
Acute heart failure	2,277	49 (8–259)	135.91 (123.53–148.30)	0.48 (0.46–0.49)	Early failure

## 4 Discussion

The medications most commonly associated with HF in the world’s largest database of AE reports were identified and categorized. The results highlight the common occurrence of this AE, as 240,050 (1.38%) of all reports involved HF. At the same time, these data underscore the seriousness of drug-related HF, as demonstrated by patient outcomes of hospitalization and death. We found that antineoplastic agents and drugs used in diabetes are associated with the largest number of HF and AHF reports; there appears to be no sex bias. From 2004 to 2023, FAERS reports of drug-induced HF increased year by year, and 52.37% of all events related to HF were in elderly patients >65 years of age; this is generally consistent with previous HF studies conducted in Europe and the United States (Butrous and Hummel, 2016; Emmons-Bell et al., 2022). Drugs is a serious threat to the patient’s life mainly by causing symptoms such as oedema and weight gain, or by inducing cardiomyocyte apoptosis leading to HF through multiple pathways. (Minotti et al., 2004; Maxwell and Jenkins, 2011; McDonagh et al., 2021). Although timely identification and drug withdrawal are the basis for clinical treatment of HF, there is still a lack of a comprehensive map of HF sensitization drugs to guide clinical practice. In this study, we introduced the main drugs related to HF from the perspective of pharmacovigilance.

Due to the lack of precise drug usage data, it is impossible to determine the actual incidence of HF caused by each medicine and compare the risk differences between different drugs. To address this issue, we introduced disproportionality analysis as an alternative method to quantify the risk of HF for each drug. Some previous studies have used this approach to study the cardiotoxicity risk of Bruton tyrosine kinase inhibitors (BTKIs), PD-1/PD-L1 inhibitors, immune checkpoint inhibitors (ICIs), as well as some single agents such as carfilzomib, doxorubicin, and hydroxychloroquine (Nishtala et al., 2020; Zhai et al., 2021; Nishiuchi et al., 2022; Wang et al., 2022; Zhai et al., 2023; Wang et al., 2024). These studies revealed the risks of some drugs causing HF, atrial fibrillation, cardiomyopathy, and so forth, to a certain extent, providing guidance for clinical practice. However, they were limited in terms of clinical application because they focused only on the cardiotoxicity risk of certain drugs or drug classes, being unable to compare HF risk between drugs and across drug classes. The statistics of the top 50 drugs with the highest proportion of reports and ATC drug categories at the SMQ level allow health professionals and other parties to quickly compare the differences in HF risk between different drugs and provide a reference for stopping patients with suspected drug-induced HF. This information can be used as a rapid tool to understand drugs and drug classes that may induce HF.

Based on the results of ADR signal detection for the 50 most frequently used drugs, the overall distribution characteristics of ADR signals were described. Specifically, we counted the sum of the positive ADR signals for each drug and found that 38 drugs had a positive ADR signal for HF, indicating a potential risk of HF. Our results suggest that significant attention should be paid to antineoplastic drugs, as well as drugs used in diabetes, antithrombotic agents, and other nervous system drugs. Among the 19 drug classes identified, the potential mechanisms by which they cause HF include: Antineoplastic agents (e.g., anthracyclines, trastuzumab), which induce cardiotoxicity through oxidative stress, mitochondrial dysfunction, and cardiomyocyte apoptosis (Destere et al., 2024); Diabetes medications such as thiazolidinediones, which can cause fluid retention and left ventricular remodeling, thereby increasing HF risk (Kendall, 2006); Antithrombotic agents, which may affect hemodynamics or lead to ischemic or hemorrhagic complications that impair cardiac function; and Nervous system drugs (e.g., antipsychotics), which may interfere with sympathetic nervous system activity and heart rate regulation. These findings emphasize the need for clinicians to closely monitor cardiac function when prescribing these drugs, particularly in high-risk populations.

In the ROR analysis of known drugs used in diabetes, rosiglitazone had the highest ROR (44.47), followed by the rosiglitazone-metformin combination (ROR 14.62), pioglitazone (ROR 6.58), metformin (ROR 1.14), and insulin lispro (ROR 0.79). Rosiglitazone and pioglitazone were initially considered beneficial for patients with HF because they can reduce blood sugar levels and reportedly have pleiotropic effects, such as reducing inflammatory markers and improving endothelial function and fibrinolysis state (Kendall, 2006). However, coadministration of rosiglitazone or pioglitazone with insulin has been shown to increase the risk of AEs such as weight gain, edema, and left ventricular hypertrophy, which can lead to HF (Henriksen et al., 2011; Giri et al., 2016). Compared to other antidiabetic drugs, metformin has been shown to be safe and to reduce the morbidity and mortality of HF (Salvatore et al., 2021). As these results are largely derived from observational studies, further evaluation should be considered for any patient diagnosed with HF after using antidiabetic drugs. Among antithrombotic agents, other nervous system drugs, antihypertensives, and other drug categories, tafamidis (ROR 11.51), macitentan (ROR 4.88), treprostinil (ROR 2.91), dabigatran (ROR 2.36), apixaban (ROR 2.34), and other drugs all had positive signals. The progress of these drug indications is closely related to HF, and we need to pay more attention to these drugs to further clarify their causal relationship. It is noteworthy that some of the drugs we identified, such as thiazolidinediones and anthracyclines, are already known to have adverse effects related to HF, such as fluid retention and cardiotoxicity, respectively. Our findings reaffirm these risks and highlight the importance of pharmacovigilance in identifying and managing drug-induced HF. Furthermore, we identified significant signal values for drugs less commonly associated with HF, such as certain nervous system drugs and antithrombotic agents, which warrant further investigation. These findings underscore the importance of re-evaluating known adverse effects in the context of real-world data and expanding our understanding of emerging risks for drugs with previously underrecognized cardiotoxicity.

AHF is one of the most common causes of hospital admission and is associated with a high risk of death (Sitkiewicz et al., 2023). Compared to chronic HF, there is less evidence on the precipitants, diagnosis, and management of AHF (Sinnenberg and Givertz, 2020; Ciccarelli et al., 2023). However, the total number of FAERS reports of AHF increased during our study period and 39.37% of patients reported to have had AHF died. We suspect that FAERS reporting bias favored more severe cases, with deaths due to HF being more likely to be reported to the FDA. Antineoplastic agents were the most reported drug class associated with AHF, followed by antithrombotic agents, immunosuppressants, and drugs used in diabetes. We found that tirofiban, metformin and saxagliptin, saxagliptin, imatinib mesylate, venlafaxine hydrochloride, atorvastatin were frequently reported in AHF. Interestingly, immunosuppressants were reported very frequently in relation to HF and AHF, but their ROR signals were negative, which may explain the false-positive results reported in individual case reports. In addition, the signal values of “Other nervous system drugs” were high (ROR 11.51 and 12.92). Of note, among the 41 drugs with positive signals in the AHF group, antineoplastic agents were overwhelmingly dominant, including doxorubicin (ROR 9.00), pembrolizumab (ROR 3.40), pembrolizumab (ROR 3.40), bortezomib (ROR 4.74), nivolumab (ROR 2.23), imatinib mesylate (ROR 2.36), carfilzomib (ROR 8.93), bevacizumab (ROR 1.54), trastuzumab (ROR 2.45), and osimertinib (ROR 7.39). With the strengthening of anti-tumor treatments and the joint application of antineoplastic drugs, the incidence of cardiac toxicity events has significantly increased. The incidence of cardiac toxicity also increases with an increased in survival time (Mercurio et al., 2016; Zhang et al., 2023). As the dose or treatment time accumulates, it can result in HF, arrhythmia, and other adverse reactions. Given the limitations and variability of the evidence published to date, clinicians must assess patients to determine whether the benefits of treatment outweigh the potential risks for each patient.

This study found that the median time to onset of drug-related HF and AHF was 83 days (IQR: 11–416) and 49 days (IQR: 8–259), respectively, with more than 50% of reported cases occurring within the first 100 days after the initiation of the drug. Previous studies of drug-induced HF have similarly found that the time interval from initiation to the onset of ADRs to HF was mostly within the first 6 months (Page et al., 2016). In WSP tests, HF and AHF had an upper limit of the 95% CI of the shape parameter <1, indicating an early failure profile, where the adverse reaction occurs mainly in the initial stages of drug use and the incidence of adverse reactions gradually decreases over time. These results suggest that the highest risk of drug-related HF is associated with the earliest phase of drug therapy.

FAERS was not able to provide summary data for more than five drugs at a time, which limited our focus to the top 50 drugs. Additionally, FAERS lacks ethnic data, and there are often inequitable outcomes in health status between different groups, with HF being no exception. Data from the United States suggest racial/ethnic differences in the incidence and survival of HF (Khera et al., 2020; Morris et al., 2022). Many FAERS reports may be confused by their indications and off-label treatments. Drugs such as sacubitril valsartan, digoxin, and furosemide are frequently reported as treatments for HF. Therefore, further studies are needed to confirm the potential for drug-induced HF identified in FAERS. Other factors that limit the applicability of FAERS data include underreporting of AEs, variation in drug lifecycle reporting, and confounding effects of concurrent medications. Nonetheless, the value of such voluntarily reported data should not be discounted, as it is a source of discovery of new underlying HF risk that might otherwise be overlooked.

Challenges in analyzing FAERS reports include the selection of relevant AE search terms and the lack of standardization of drug names in the database. We addressed name standardization by manually entering generic drug names for all brand names, international names, and misspelled names. Due to the practical work required to standardize drug names and determine the frequency of AEs from the database, only RORs were calculated for the 50 drugs with the highest reported frequency in each category.

Despite its limitations, this study highlights the common occurrence of HF and provides information on the most commonly reported medications associated with HF. Strengths of this study include the utilization of a dataset of AEs reported over the last 20 years. Although there is no consensus on which data mining algorithm is most effective for detecting ADR associations, we selected ROR based on its simplicity and data showing higher sensitivity.

## 5 Conclusion

Drug-induced HF is a life-threatening drug safety issue of deep concern to cardiologists, pharmacists, drug manufacturers, and regulatory agencies. This study reviewed the reports of adverse reactions related to HF in the FAERS database, summarized the list of potential HF-causing drugs and their corresponding proportion of AE reports, and detected their ADR signals at the SMQ and PT levels. To the best of our knowledge, this study is the first to provide a picture of the current culprit drugs for HF from a pharmacovigilance perspective. Although our study has some inherent limitations due to its nature, our results can, to a certain extent, serve as reference information for patients, clinical providers, regulatory agencies, and others who are concerned about drug cardiotoxicity and thereby help improve clinical practice.

## Data Availability

Publicly available datasets were analyzed in this study. This data can be found here: https://www.fda.gov/drugs/fdas-adverse-event-reporting-system-faers/potential-signals-serious-risksnew-safety-information-identified-fda-adverse-event-reporting-system.
