# Supplementary material for: Drug-induced heart failure: a real-world pharmacovigilance study using the FDA adverse event reporting system database
Source: Front Pharmacol. 2025 Jan 15;15:1523136. doi: 10.3389/fphar.2024.1523136 (PMC11775474; doi:10.3389/fphar.2024.1523136)
Supplement: Supplementary file 1 [file DataSheet1.docx]

**Additional file title: Drug-induced heart failure: A real-world pharmacovigilance study using the FDA Adverse Event Reporting System database**

**Table S1**. PTs contained in the narrow-scope search of “Cardiac failure (SMQ)”

| PT | MedDRA code |
| --- | --- |
| Acute left ventricular failure | 10063081 |
| Acute pulmonary oedema | 10001029 |
| Acute right ventricular failure | 10063082 |
| Cardiac asthma | 10007522 |
| Cardiac failure | 10007554 |
| Cardiac failure acute | 10007556 |
| Cardiac failure chronic | 10007558 |
| Cardiac failure congestive | 10007559 |
| Cardiac failure high output | 10007560 |
| Cardiogenic shock | 10007625 |
| Cardiohepatic syndrome | 10082480 |
| Cardiopulmonary failure | 10051093 |
| Cardiorenal syndrome | 10068230 |
| Chronic left ventricular failure | 10063083 |
| Chronic right ventricular failure | 10063084 |
| Congestive hepatopathy | 10084058 |
| Cor pulmonale | 10010968 |
| Cor pulmonale acute | 10010969 |
| Cor pulmonale chronic | 10010970 |
| Ejection fraction decreased | 10050528 |
| Hepatojugular reflux | 10051448 |
| Left ventricular failure | 10024119 |
| Low cardiac output syndrome | 10024899 |
| Neonatal cardiac failure | 10049780 |
| Obstructive shock | 10073708 |
| Pulmonary oedema | 10037423 |
| Pulmonary oedema neonatal | 10050459 |
| Radiation associated cardiac failure | 10076203 |
| Right ventricular ejection fraction decreased | 10075337 |
| Right ventricular failure | 10039163 |
| Ventricular failure | 10060953 |
| Cardiac failure (SMQ)^†^ | 20000004 |

^†^This is an SMQ term that includes the aforementioned 31 Preferred Terms in narrow-scope search. **Abbreviations:** PT, preferred term; MedDRA, Medical Dictionary for Drug Regulatory Activities; SMQ, Standardized MedDRA Query.

**Table S2**. Fourfold table of measures of disproportionality.

|  | Drug of interest | Other drugs | Total |
| --- | --- | --- | --- |
| AE of interest | a | b | a+b |
| Other adverse events | c | d | c+d |
| Total | a+c | b+d | a+b+c+d |

Note: ROR＝(a/c)/(b/d), 95% CI=e^ln^^(ROR)±1.96(1/a+1/b+1/c+1/d)^.05^. **Abbreviations:** AE, adverse event; ROR, reporting odds ratio.

**Table S3**. Top 50 drugs associated with heart failure.

| Top 50 Drug name | Heart failure event reports | Total adverse event reports | ROR (95%Cl) | ATC drug classes |
| --- | --- | --- | --- | --- |
| Rosiglitazone | 27378 | 177241 | 44.47 (43.87–45.08) | A10BD03 |
| Sacubitril valsartan | 8847 | 275424 | 7.41 (7.26–7.58) | C09DX04 |
| Adalimumab | 4524 | 1858944 | 0.52 (0.5–0.53) | L04AB04 |
| Ambrisentan | 4341 | 209237 | 4.65 (4.51–4.79) | C02KX02 |
| Rofecoxib | 4232 | 240024 | 3.93 (3.81–4.06) | M01AH02 |
| Lenalidomide | 3672 | 660442 | 1.21 (1.17–1.25) | L04AX04 |
| Macitentan | 3567 | 163710 | 4.88 (4.71–5.04) | C02KX04 |
| Bosentan | 3536 | 125541 | 6.35 (6.14–6.56) | C02KX01 |
| Treprostinil | 3432 | 261057 | 2.91 (2.81–3.01) | B01AC21 |
| Apixaban | 2560 | 240196 | 2.34 (2.25–2.44) | B01AF02 |
| Trastuzumab | 2387 | 116287 | 4.57 (4.39–4.76) | L01XC03 |
| Etanercept | 1922 | 1358886 | 0.3 (0.29–0.31) | L04AB01 |
| Amlodipine | 1740 | 174674 | 2.18 (2.08–2.29) | C08CA01 |
| Rituximab | 1699 | 441412 | 0.82 (0.78–0.86) | L01XC02 |
| Nilotinib | 1677 | 89525 | 1.48 (1.37–1.61) | L01XE08 |
| Ibrutinib | 1573 | 198839 | 1.73 (1.65–1.82) | L01EL01 |
| Digoxin | 1573 | 78824 | 4.43 (4.21–4.65) | C01AA05 |
| Dabigatran | 1552 | 144320 | 2.36 (2.24–2.48) | B01AE07 |
| Celecoxib | 1496 | 105293 | 1.74 (1.62–1.86) | L01XX33  M01AH01 |
| Rivaroxaban | 1478 | 258780 | 1.24 (1.18–1.31) | B01AF01 |
| Sildenafil | 1404 | 98138 | 3.15 (2.99–3.32) | G04BE03 |
| Iloprost | 1385 | 21199 | 5.94 (5.47–6.46) | B01AC11 |
| Valsartan | 1385 | 119061 | 2.08 (1.96–2.21) | C09CA03 |
| Infliximab | 1204 | 743712 | 0.35 (0.33–0.37) | L04AB02 |
| Dasatinib | 1201 | 61091 | 4.35 (4.11–4.61) | L01XE06 |
| Interferon beta-1a | 1171 | 478043 | 0.53 (0.5–0.56) | L03AB07 |
| Rosiglitazone-metformin combination | 1154 | 8704 | 14.62 (13.41–15.94) | A10BD03 |
| Denosumab | 1133 | 307392 | 0.5 (0.46–0.54) | M05BX04 |
| Epoprostenol | 1111 | 79420 | 3.86 (3.66–4.07) | B01AC09 |
| Selexipag | 1062 | 64868 | 3.61 (3.4–3.84) | B01AC27 |
| Insulin lispro | 996 | 196111 | 0.79 (0.73–0.85) | A10AB04  A10AC04  A10AD04 |
| Teriparatide | 989 | 348077 | 0.61 (0.58–0.65) | H05AA02 |
| Pregabalin | 987 | 364777 | 0.59 (0.55–0.62) | N03AX16 |
| Sunitinib | 985 | 137189 | 1.57 (1.47–1.67) | L01XE04 |
| Pomalidomide | 982 | 140999 | 1.12 (1.04–1.21) | L04AX06 |
| Palbociclib | 880 | 244730 | 0.6 (0.55–0.64) | L01EF01 |
| Tafamidis | 837 | 22075 | 11.51 (10.84–12.23) | N07XX08 |
| Doxorubicin | 806 | 87155 | 4.32 (4.12–4.54) | L01DB01 |
| Thalidomide | 792 | 60445 | 2.25 (2.08–2.44) | L04AX02 |
| Paricalcitol | 712 | 21108 | 5.78 (5.31–6.29) | H05BX02 |
| Furosemide | 710 | 73351 | 4.52 (4.3–4.76) | C03CA01 |
| Bortezomib | 674 | 86010 | 2.54 (2.39–2.7) | L01XX32 |
| Bevacizumab | 622 | 195652 | 1.09 (1.02–1.16) | L01XC07 |
| Nivolumab | 609 | 166605 | 1.15 (1.08–1.23) | L01FF01 |
| Tiotropium bromide | 607 | 148664 | 1.13 (1.05–1.21) | R03BB04 |
| Clozapine | 571 | 232463 | 1.08 (1.02–1.14) | N05AH02 |
| Eculizumab | 566 | 150182 | 0.88 (0.81–0.95) | L04AA25 |
| Pioglitazone | 550 | 31555 | 6.58 (6.16–7.02) | A10BG03 |
| Metformin | 548 | 219756 | 1.14 (1.07–1.21) | A10BA02 |
| Oxycodone | 540 | 534816 | 0.32 (0.3–0.35) | N02AA05 |

**Abbreviations:** ATC, anatomical therapeutic chemical; CI, confidence interval.

**Table S4**. Top 50 drugs associated with heart failure–ATC drug classes

| Drug class | Heart failure event reports | Total adverse event reports | ROR (95%Cl) |
| --- | --- | --- | --- |
| [A10 Drugs used in diabetes](https://atcddd.fhi.no/atc_ddd_index/?code=A10&showdescription=no) | 30717 | 602137 | 12.51 (12.35–12.66) |
| L01 Antineoplastic agents | 14500 | 1913776 | 1.68 (1.65–1.71) |
| L04 Immunosuppressants | 13279 | 4960079 | 0.55 (0.54–0.56) |
| C02 Antihypertensives | 12935 | 622213 | 4.7 (4.61–4.78) |
| B01 Antithrombotic agents | 12035 | 1057553 | 2.54 (2.49–2.59) |
| C09 Agents acting on the renin-angiotensin system | 9980 | 384253 | 5.82 (5.7–5.94) |
| M01 Antiinflammatory and antirheumatism products | 5069 | 339996 | 3.27 (3.18–3.37) |
| C08 Calcium channel blockers | 1740 | 172934 | 2.18 (2.08–2.29) |
| C01 Cardiac therapy | 1573 | 77251 | 4.43 (4.21–4.65) |
| H05 Calcium homeostasis | 1537 | 367648 | 0.9 (0.86–0.95) |
| C03 Diuretics | 1496 | 71855 | 4.52 (4.3–4.76) |
| G04 Urologicals | 1404 | 96734 | 3.15 (2.99–3.32) |
| L03 Immunostimulants | 1171 | 476620 | 0.53 (0.5–0.56) |
| N05 Psycholeptics | 1154 | 231309 | 1.08 (1.02–1.14) |
| N07 Other nervous system drugs | 1111 | 20964 | 11.51 (10.84–12.23) |
| N03 Antiepileptics | 987 | 363790 | 0.59 (0.55–0.62) |
| N02 Analgesics | 806 | 533758 | 0.32 (0.3–0.35) |
| R03 Drugs for obstructive airway disease | 773 | 147639 | 1.13 (1.06–1.22) |
| M05 Drugs for the treatment of bone diseases | 710 | 306430 | 0.5 (0.46–0.54) |

**Table S5**. Clinical characteristics of reports from the FAERS database (2004 to 2023).

| Characteristics | Drug-related acute heart failure (N = 5971) |
| --- | --- |
| Number | 5971 |
| Sex | |
| Male | 2559 (42.9%) |
| Female | 2791 (46.7%) |
| Unknown | 621 (10.4%) |
| Age | |
| < 18 | 134 (2.2%) |
| 18 ≥ and <65 | 2002 (33.5%) |
| 65 ≥ and <85 | 2208 (37.0%) |
| ≥ 85 | 304 (5.1%) |
| Unknown | 1323 (22.2%) |
| Weight | |
| < 50 | 357 (6.0%) |
| 50 ≥ and <100 | 1521 (25.5%) |
| ＞ 100 | 264 (4.4%) |
| Unknown | 3829 (64.1%) |
| Occupation of the reporter^a^ | |
| Healthcare professional | 4911 (82.2%) |
| Non-healthcare professional | 735 (12.3%) |
| Unknown | 325 (5.4%) |
| Reporting year | |
| 2004 | 87 (1.5%) |
| 2005 | 102 (1.7%) |
| 2006 | 134 (2.2%) |
| 2007 | 88 (1.5%) |
| 2008 | 109 (1.8%) |
| 2009 | 120 (2%) |
| 2010 | 143 (2.4%) |
| 2011 | 88 (1.5%) |
| 2012 | 170 (2.8%) |
| 2013 | 203 (3.4%) |
| 2014 | 247 (4.1%) |
| 2015 | 318 (5.3%) |
| 2016 | 342 (5.7%) |
| 2017 | 400 (6.7%) |
| 2018 | 483 (8.1%) |
| 2019 | 619 (10.4%) |
| 2020 | 581 (9.7%) |
| 2021 | 543 (9.1%) |
| 2022 | 605 (10.1%) |
| 2023 | 589 (9.9%) |
| Outcome^b^ | |
| Death | 2351 (39.4%) |
| Hospitalization | 2112 (35.4%) |
| Life-threatening | 696 (11.7%) |
| Other Serious Outcome | 602 (10.1%) |
| Disability | 9 (0.2%) |
| Unknown | 201 (3.5%) |

^a^Healthcare professionals include reporters such as physicians and pharmacists; nonhealthcare professionals include reporters such as consumers and lawyers.

^b^Because a case may experience different clinical outcomes during drug therapy, it is reasonable to expect that the sum percentage of the outcome under this item may exceed 100%.

**Table S6**. Top 50 drugs associated with acute heart failure.

| Top 50 Drug name | Acute heart failure event reports | ROR (95%Cl) | PT:10007556 event reports | ROR (95%Cl) | PT:10063081 event reports | ROR (95%Cl) | PT:10063082 event reports | ROR (95%Cl) | ATC drug classes |
| --- | --- | --- | --- | --- | --- | --- | --- | --- | --- |
| Sacubitril and valsartan | 179 | 5.73 (4.93–6.64) | 164 | 5.96 (5.1–6.96) | 15 | 4.98 (2.98–8.32) | / | / | C09DX04 |
| Macitentan | 112 | 5.97 (4.95–7.2) | 88 | 5.31 (4.3–6.56) | 14 | 7.83 (4.61–13.3) | 10 | 25.01 (13.14–47.61) | C02KX04 |
| Tirofiban | 102 | 266.77 (218.71–325.39) | 102 | 303.3 (248.6–370.03) | / | / | / | / | B01AC17 |
| Treprostinil | 101 | 3.36 (2.76–4.09) | 77 | 2.9 (2.32–3.63) | 2 | 0.69 (0.17–2.75) | 22 | 38.03 (24.1–60.02) | B01AC21 |
| Doxorubicin | 90 | 9 (7.31–11.08) | 79 | 8.96 (7.17–11.19) | 11 | 11.51 (6.34–20.9) | / | / | L01DB01 |
| Ambrisentan | 83 | 3.44 (2.77–4.27) | 65 | 3.05 (2.39–3.9) | 8 | 3.46 (1.72–6.95) | 10 | 19.55 (10.27–37.22) | C02KX02 |
| Bosentan | 80 | 5.54 (4.44–6.9) | 65 | 5.1 (3.99–6.51) | 6 | 4.32 (1.93–9.65) | 9 | 29.14 (14.83–57.3) | C02KX01 |
| Rivaroxaban | 74 | 2.47 (1.97–3.11) | 59 | 2.24 (1.73–2.89) | 11 | 3.86 (2.13–7.02) | 4 | 6.03 (2.23–16.31) | B01AF01 |
| Lenalidomide | 73 | 0.95 (0.75–1.2) | 66 | 0.97 (0.76–1.24) | 7 | 0.95 (0.45–2) | / | / | L04AX04 |
| Adalimumab | 67 | 0.3 (0.24–0.38) | 54 | 0.28 (0.21–0.36) | 11 | 0.52 (0.29–0.95) | 2 | 0.4 (0.1–1.62) | L04AB04 |
| Furosemide | 63 | 7.45 (5.81–9.55) | 62 | 8.33 (6.48–10.7) | 1 | 1.22 (0.17–8.69) | / | / | C03CA01 |
| Venlafaxine hydrochloride | 59 | 3.16 (2.44–4.08) | 54 | 3.28 (2.51–4.29) | 5 | 2.79 (1.16–6.73) | / | / | N06AX16 |
| Atorvastatin | 59 | 2.26 (1.75–2.92) | 40 | 1.73 (1.27–2.37) | 18 | 7.35 (4.6–11.75) | 1 | 1.69 (0.24–12.1) | C10AA05 |
| Dabigatran | 50 | 2.99 (2.27–3.95) | 48 | 3.26 (2.45–4.33) | 2 | 1.24 (0.31–4.98) | / | / | B01AE07 |
| Amlodipine | 50 | 2.47 (1.87–3.26) | 42 | 2.35 (1.74–3.19) | 8 | 4.15 (2.06–8.33) | / | / | C08CA01 |
| Clozapine | 50 | 1.85 (1.4–2.45) | 43 | 1.81 (1.34–2.44) | 7 | 2.72 (1.29–5.73) | / | / | N05AH02 |
| Pembrolizumab | 48 | 3.4 (2.56–4.52) | 44 | 3.54 (2.63–4.76) | 4 | 2.95 (1.11–7.9) | / | / | L01FF02 |
| Apixaban | 48 | 1.72 (1.3–2.29) | 45 | 1.83 (1.37–2.46) | 3 | 1.12 (0.36–3.48) | / | / | B01AF02 |
| Bortezomib | 47 | 4.74 (3.56–6.31) | 44 | 5.03 (3.74–6.77) | 3 | 3.15 (1.01–9.78) | / | / | L01XG01 |
| Rituximab | 46 | 0.89 (0.67–1.2) | 42 | 0.93 (0.68–1.26) | 3 | 0.61 (0.2–1.89) | 1 | 0.86 (0.12–6.16) | L01FA01 |
| Methotrexate | 46 | 0.82 (0.62–1.1) | 45 | 0.91 (0.68–1.23) | 1 | 0.19 (0.03–1.32) | / | / | L01BA01 L04AX03 |
| Amiodarone | 45 | 5.27 (3.93–7.07) | 41 | 5.45 (4.01–7.41) | 3 | 3.66 (1.18–11.37) | 1 | 5.19 (0.73–37.1) | C01BD01 |
| Metformin and saxagliptin | 43 | 126.94 (93.84–171.72) | 8 | 26.33 (13.15–52.72) | 35 | 1135.88 (805.54–1601.67) | / | / | A10BD10 |
| Nivolumab | 43 | 2.23 (1.65–3.01) | 42 | 2.47 (1.82–3.35) | 1 | 0.54 (0.08–3.83) | / | / | L01FF01 |
| Imatinib mesylate | 43 | 2.36 (1.75–3.18) | 43 | 2.68 (1.98–3.61) | / | / | / | / | L01EA01 |
| Valsartan | 42 | 3.05 (2.25–4.13) | 40 | 3.3 (2.42–4.5) | 2 | 1.51 (0.38–6.05) | / | / | C09CA03 |
| Tacrolimus | 42 | 2.06 (1.52–2.79) | 37 | 2.06 (1.49–2.84) | 3 | 1.53 (0.49–4.76) | 2 | 4.38 (1.08–17.68) | D11AH01 L04AD02 |
| Clopidogrel | 41 | 3.06 (2.25–4.16) | 25 | 2.11 (1.43–3.13) | 9 | 7.08 (3.66–13.67) | 7 | 24.25 (11.34–51.89) | B01AC04 |
| Carfilzomib | 37 | 8.93 (6.46–12.34) | 33 | 9.04 (6.42–12.73) | 4 | 10.07 (3.77–26.94) | / | / | L01XG02 |
| Metoprolol | 37 | 3.59 (2.6–4.95) | 36 | 3.96 (2.85–5.5) | 1 | 1.01 (0.14–7.16) | / | / | C07AB02 |
| Bevacizumab | 35 | 1.54 (1.11–2.15) | 31 | 1.55 (1.09–2.21) | 3 | 1.38 (0.44–4.28) | 1 | 1.96 (0.27–13.98) | L01FG01 S01LA08 |
| Selexipag | 34 | 4.52 (3.23–6.34) | 28 | 4.22 (2.91–6.12) | 5 | 6.96 (2.89–16.78) | 1 | 5.91 (0.83–42.23) | B01AC27 |
| Rosiglitazone | 34 | 1.65 (1.18–2.31) | 33 | 1.82 (1.29–2.56) | 1 | 0.5 (0.07–3.59) | / | / | A10BG02 |
| Saxagliptin | 34 | 43.91 (31.32–61.56) | 10 | 14.54 (7.82–27.05) | 24 | 334.89 (222.41–504.27) | / | / | A10BH03 |
| Trastuzumab | 33 | 2.45 (1.74–3.44) | 32 | 2.69 (1.9–3.81) | 1 | 0.77 (0.11–5.48) | / | / | L01FD01 |
| Tafamidis | 33 | 12.92 (9.18–18.2) | 29 | 12.88 (8.94–18.56) | 4 | 16.35 (6.11–43.71) | / | / | N07XX08 |
| Osimertinib | 32 | 7.39 (5.22–10.47) | 30 | 7.87 (5.49–11.26) | 2 | 4.81 (1.2–19.28) | / | / | L01EB04 |
| Infliximab | 32 | 0.37 (0.26–0.52) | 18 | 0.23 (0.15–0.37) | 14 | 1.7 (1–2.9) | / | / | L04AB02 |
| Ticagrelor | 31 | 5.52 (3.88–7.86) | 28 | 5.66 (3.9–8.2) | 3 | 5.57 (1.79–17.33) | / | / | B01AC24 |
| Riociguat | 30 | 5.5 (3.84–7.88) | 23 | 4.78 (3.17–7.2) | 5 | 9.6 (3.98–23.15) | 2 | 16.41 (4.06–66.29) | C02KX05 |
| Prednisone | 30 | 2.46 (1.72–3.52) | 27 | 2.51 (1.72–3.66) | 3 | 2.56 (0.82–7.97) | / | / | A07EA03 H02AB07 |
| Ibrutinib | 28 | 1.21 (0.84–1.75) | 23 | 1.13 (0.75–1.7) | 5 | 2.26 (0.94–5.46) | / | / | L01EL01 |
| Epoprostenol | 28 | 3.05 (2.1–4.42) | 16 | 1.97 (1.21–3.22) | / | / | 12 | 63.16 (34.93–114.22) | B01AC09 |
| Bisoprolol | 26 | 5.24 (3.56–7.7) | 24 | 5.48 (3.67–8.19) | 2 | 4.2 (1.05–16.82) | / | / | C07AB07 |
| Metformin | 26 | 1.02 (0.69–1.5) | 22 | 0.98 (0.64–1.48) | 4 | 1.64 (0.61–4.38) | / | / | A10BA02 |
| Rofecoxib | 25 | 0.89 (0.6–1.32) | 22 | 0.89 (0.59–1.36) | 3 | 1.12 (0.36–3.49) | / | / | M01AH02 |
| Etanercept | 25 | 0.15 (0.1–0.23) | 24 | 0.17 (0.11–0.25) | 1 | 0.06 (0.01–0.46) | / | / | L04AB01 |
| Darbepoetin alfa | 24 | 3.29 (2.2–4.91) | 23 | 3.58 (2.38–5.39) | 1 | 1.43 (0.2–10.14) | / | / | B03XA02 |
| Oseltamivir | 23 | 5.75 (3.82–8.66) | 23 | 6.53 (4.33–9.83) | / | / | / | / | J05AH02 |
| Digoxin | 21 | 2.29 (1.49–3.52) | 21 | 2.6 (1.7–4) | / | / | / | / | C01AA05 |

**Table S7**. Top 50 drugs associated with acute heart failure–ATC drug classes

| Drug class | Acute cardiac failure event reports | Total adverse event reports | ROR (95%Cl) |
| --- | --- | --- | --- |
| L01 Antineoplastic agents | 528 | 2122432 | 2.24 (2.05–2.45) |
| B01 Antithrombotic agents | 509 | 1215319 | 3.83 (3.5–4.2) |
| L04 Immunosuppressants | 285 | 5277304 | 0.44 (0.39–0.49) |
| A10 Drugs used in diabetes | 137 | 406303 | 2.94 (2.48–3.48) |
| C02 Antihypertensives | 305 | 545232 | 5.01 (4.46–5.62) |
| C07 Beta blocking agents | 63 | 131807 | 4.14 (3.23–5.3) |
| A07 Antidiarrheals, intestinal antiinflammatory/antiinfective agents | 30 | 105176 | 2.46 (1.72–3.52) |
| B03 Antianemic preparations | 24 | 62831 | 3.29 (2.2–4.91) |
| C01 Cardiac therapy | 66 | 152568 | 3.75 (2.94–4.77) |
| C03 Diuretics | 63 | 73288 | 7.45 (5.81–9.55) |
| C08 Calcium channel blockers | 50 | 174624 | 2.47 (1.87–3.26) |
| C09 Agents acting on the renin-angiotensin system | 221 | 394012 | 4.96 (4.34–5.68) |
| C10 Lipid-modifying agents | 59 | 225547 | 2.26 (1.75–2.92) |
| D11 Other dermatological preparations | 42 | 175969 | 2.06 (1.52–2.79) |
| H02 Corticosteroids for systemic use | 30 | 105176 | 2.46 (1.72–3.52) |
| J05 Antivirals for systemic use | 23 | 34460 | 5.75 (3.82–8.66) |
| M01 Antiinflammatory and antirheumatic products | 25 | 239999 | 0.89 (0.6–1.32) |
| N05 Psycholeptics | 50 | 232413 | 1.85 (1.4–2.45) |
| N06 Psychoanaleptics | 59 | 161490 | 3.16 (2.44–4.08) |
| N07 Other nervous system drugs | 33 | 22042 | 12.92 (9.18–18.2) |
| S01 Ophthalmologicals | 35 | 195365 | 1.54 (1.11–2.15) |


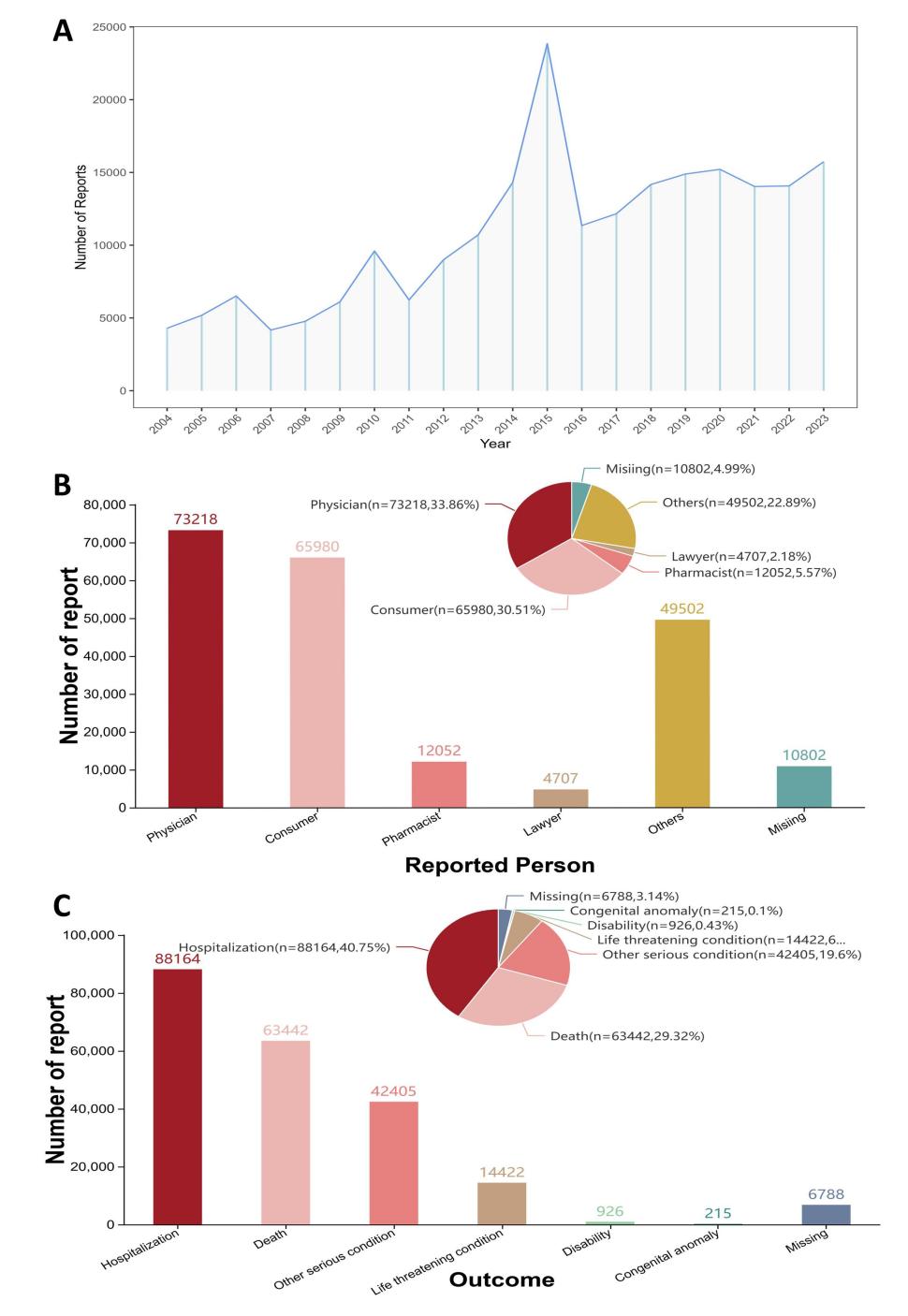


Figure S1 (A) The number of annual adverse reaction reports. (B) the outcome distribution of adverse reactions in patients. (C) The occupational distribution of the reporter.


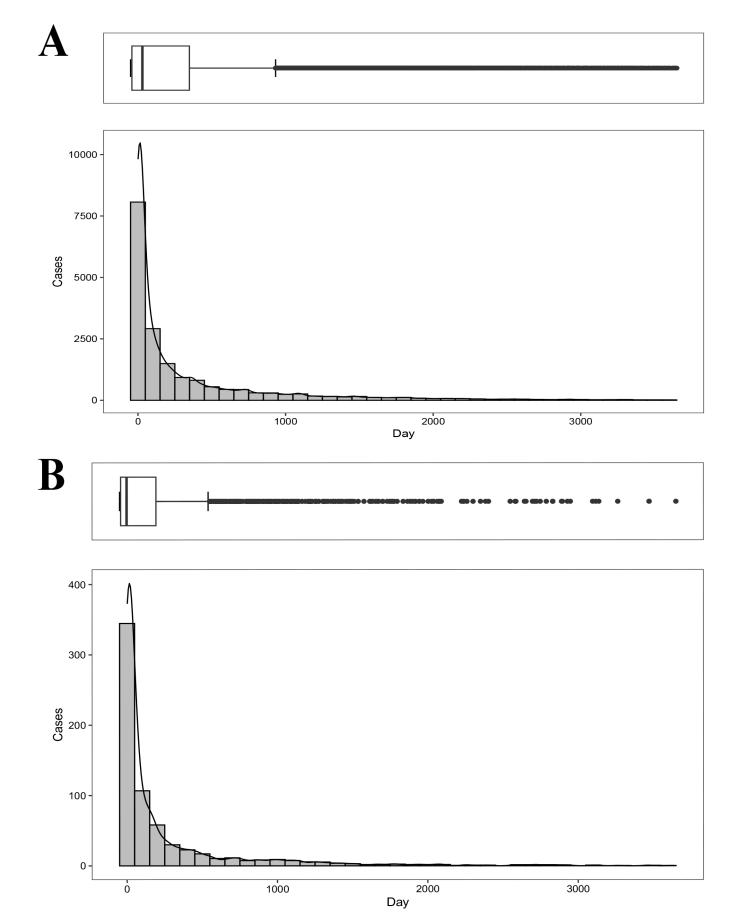


Figure S2. Histograms of (A) heart failure and (B) acute heart failure.
